# First Experimental Evidence of Anti-Stokes Laser-Induced Fluorescence Emission in Microdroplets and Microfluidic Systems Driven by Low Thermal Conductivity of Fluorocarbon Carrier Oil

**DOI:** 10.3390/mi14040765

**Published:** 2023-03-29

**Authors:** Zain Hayat, Abdel El Abed

**Affiliations:** Laboratoire Lumière Matière et Interfaces (LUMIN), UMR 9024, Ecole Normale Supérieure Paris Saclay, CentraleSupélec, CNRS, Université Paris-Saclay, 4 Avenue des Sciences, 91190 Gif-sur-Yvette, France

**Keywords:** droplets, microfluidics, LIF (laser-induced fluorescence), SF (stokes fluorescence), ASF (anti-stokes fluorescence), thermal sensitivity, low thermal conductivity, TICT (twisted intermolecular charge transfer)

## Abstract

With the advent of many optofluidic and droplet microfluidic applications using laser-induced fluorescence (LIF), the need for a better understanding of the heating effect induced by pump laser excitation sources and good monitoring of temperature inside such confined microsystems started to emerge. We developed a broadband highly sensitive optofluidic detection system, which enabled us to show for the first time that Rhodamine-B dye molecules can exhibit standard photoluminescence as well as blue-shifted photoluminescence. We demonstrate that this phenomenon originates from the interaction between the pump laser beam and dye molecules when surrounded by the low thermal conductive fluorocarbon oil, generally used as a carrier medium in droplet microfluidics. We also show that when the temperature is increased, both Stokes and anti-Stokes fluorescence intensities remain practically constant until a temperature transition is reached, above which the fluorescence intensity starts to decrease linearly with a thermal sensitivity of about −0.4%/°C for Stokes emission or −0.2%/°C for anti-Stokes emission. For an excitation power of 3.5 mW, the temperature transition was found to be about 25 °C, whereas for a smaller excitation power (0.5 mW), the transition temperature was found to be about 36 °C.

## 1. Introduction

It is well known, since the early report of J. de Kowalski in 1910 [1], that many dyes can emit fluorescence at wavelengths shorter than the excitation’s. Through this process, named Anti-Stokes Fluorescence (ASF) by opposition to the standard Stokes Fluorescence (SF), the excitation of electrons occurs from thermally excited levels of the ground state $S_{0}$ to the first excited electronic state $S_{1}$ (hot band absorption) [2,3,4,5,6,7,8,9,10]. In the particular case of laser-induced fluorescence (LIF), the excitation of dye molecules with a pump laser generally results in heat generation in the medium. The lost difference between the pump and emitted photons is transformed to heat and results, in turn, in an increase in the temperature of the medium and to the emission of ASF, thanks to an enhancement of the phonons of the excited dye’s atoms. Because the population of thermally excited levels of the ground state is temperature dependent, ASF intensity depends exponentially on temperature following a Boltzmann distribution. Therefore, ASF may be used efficiently to measure local temperature variations at the micro and nanoscale [11,12]. Furthermore, since this process involves the emission of higher energy photons than those which are absorbed, ASF can cause the removal of energy from the material illuminated and consequently may lead to its refrigeration [13]. The idea of using anti-Stokes fluorescence to cool solid-state matter was first proposed by Pringsheim in 1929 [9,10].

With the development of many microfluidics’ applications, the need for good monitoring of temperature inside micro-sized reactors and a better understanding of the heating effect induced by pump laser excitation sources inside such confined microsystems started to emerge. In droplet-based microfluidic technology, in particular, water-in-oil microdroplets (W/O) are usually dispersed in fluorocarbon oils. These fluorocarbon oils are bio-compatible and chemically inert, and they do not interact with droplet contents nor swell the microfluidic device substrates.

In this work, we report the first experimental evidence of anti-Stokes fluorescence emission from rhodamine B dye in microfluidic systems, which is induced by the confinement of heat induced by a pump laser in flowing microdroplets carried inside microchannels with a fluorocarbon oil. This achievement was enabled by the improvement brought to our dual channel optical setup [14], based on the use of an off-axis parabolic mirror, which allows for a highly sensitive and broadband detection of the fluorescence signals emitted by fluorescent droplets carried by the continuous fluorocarbon oil phase. We show, in particular, that droplets’ fluorescence is a thermally activated process based on a hot band absorption of Rhodamine B molecules at the fluorocarbon oil droplet interface and correlate the observation of such a phenomenon to the very small thermal conductivity value of the used fluorocarbon oil, about 0.065 W/m·K. This value is of the same order as the thermal conductivity of air and is an order smaller than water’s thermal conductivity (0.614 W/m·K). We also show for the first time that the balance between the linear decrease in the fluorescence of Rhodamine B when increasing temperature (with a thermal sensitivity -slope- of about −1%/°C) and the thermally activated anti-Stokes fluorescence leads to an interesting transition in the fluorescence emission properties of rhodamine B vs. temperature, which depend on illumination and heating/cooling flow rates. Typically, RhB fluorescence intensity decreases with an increase in temperature and a thermal sensitivity of *S*∼2%/°C (in the 20–50 °C temperature range), which is an order of magnitude higher than the thermal sensitivity of most other fluorescence temperature-dependent dyes [15,16]. Thermal sensitivity (*S*) of the temperature-sensitive dye is defined as the quotient of the change in the fluorescence intensity of the dye and the change in the temperature value. It is expressed in (% change in fluorescence/K, (or/°C). The high thermal sensitivity of RhB makes it one of the best molecular probes for performing temperature measurements at the microscopic level. Nevertheless, using dyes for probing the temperature locally is not straightforward, mainly because of inherent fluctuations in the intensity of the excitation laser source and local fluctuations of dye concentration in the solution. These issues make the calibration of the LIF-dependence of dyes vs. temperature practically very difficult. To avoid such problems, Sakakibara et al. [15] and Ebert et al. [16] used two dyes, which absorb at the same excitation wavelength but emit fluorescence at different wavelengths. They used, for instance, Rhodamine 110, whose fluorescence depends very weakly on temperature, as a reference for the calibration of the LIF of RhB.

The temperature sensitivity *S* of fluorescence emission of Rhodamine B is generally explained by the existence of a photoinduced reaction leading to the formation of a highly polar excited conformer called a “twisted intermolecular charge transfer” state (acronym TICT) [17,18,19]. More exactly, the excitation causes a difference between the charge distributions in the excited singlet state and in the singlet ground state. Therefore, a reorganization of the solvent molecules and a slight conformational change in the fluorophore may occur. This sequence of events further leads the excited molecule to a state with increased charge transfer and a distortion of its geometry. Since such distortion is thermally activated, the rate of formation of the TICT state increases with temperature, and the final result is a reduction in the quantum yield of rhodamine B molecules with increasing temperature [17].

## 2. Materials and Methods

### 2.1. Broadband Reflection Mode Optofluidic Setup

**Figure 1:**
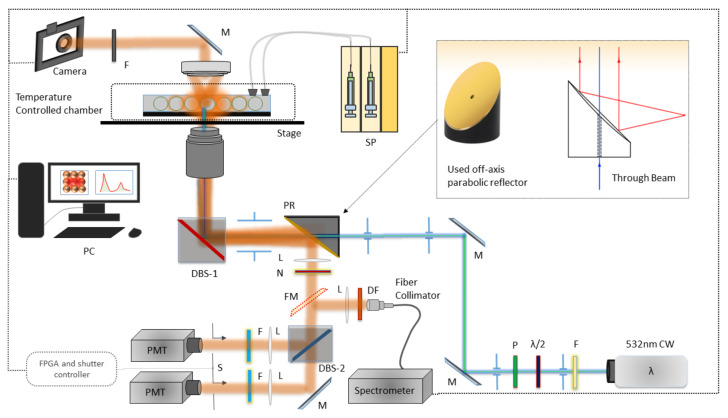
Reflection mode-dual channel microfluidic droplet monitoring setup, consisting of a continuous wave (CW) 532 nm laser source. Laser is band limited by a bandpass filter followed by a half-wave plate and a polarizer. After couple of mirrors (M) and iris diaphragms, laser is coupled from rear end of the off-axis reflector (inset: used Thorlabs Off-Axis parabolic mirror with a hole parallel to collimated laser beam). Later, laser is reflected by DBS-1 (735 nm Brightline multiphoton single-edge dichroic beamsplitter: FF735-Di02-25x36), coupled into the microscope objective, and hits the sample. The fluorescent signal is coupled back to the objective, then hits the 735 nm edge filter, and gets reflected from the parabolic mirror. A laser notch filter (N) is placed inline to cut off any stray laser component present in the reflected signal. Another dichroic beamsplitter, DBS-2 (532 nm Brightline single-edge laser dichroic beamsplitter: Di02-R532-25x36), splits the fluorescent signal into two bands, one below 540 nm and one above 540 nm. The two components of the fluorescence signals emitted by the dye are separated, filtered, and collected on two different photo-multiplier tubes (PMT). For spectral investigations, a flipping mirror (FM), a lens (L), a density filter (DF), and an air-spaced fiber collimator was used to couple the signal to the spectrometer.

The original results presented in this study were obtained thanks to a crucial enhancement of our optical detection system by the introduction of an off-axis parabolic reflector (PR) with a centred hole in the experimental setup. Enhancement enables an efficient collection of the emitted luminescence of the droplets spatially and spectrally, i.e., over a large spectral band, including wavelengths that are shorter than the excitation wavelength. The used off-axis parabolic mirror has been developed by Thorlabs to allow a collinear beam to pass through the mirror into the collimated reflection of a point source, as illustrated in the inset of Figure 1. The diameter of the hole is 0.13 inch (3.2 mm), which is just large enough to accept a laser beam input and does not alter the reflection properties of the parabolic mirror. This enables the incident excitation laser beam to hit the sample without the need to use any cut-off dichroic filter and to collect a large amount of emitted fluorescence from the sample. The system discussed in previous studies required active changes whenever a different fluorescent dye was used in the experiment [14,20]. To make the system compatible with each of them, modifications were eminent. To account for the system improvement, we developed a novel reflection mode dual channel detection system. The system design involved three parts: the source section housed laser with all of lumped components, for instance, half/quarter wave retarders, filters, and collimators. Secondly, the droplet generation, incubation, and visual inspection region, and finally the detection mode in reflected signal. Now we look at the different units of the system.

For fluorescent excitation, we employed an inverted stage X-73 Olympus microscope (Olympus France, Rungis, France)for droplet inspection. As the microscope assembly housed only transmission mode setup, to solve for acquiring the complete signal at reflection, a combination of two dichroic mirrors and one off-axis parabolic reflector were incorporated. Laser light passed through the small 3.2 mm aperture tube inside the off-axis parabolic reflector, got reflected by the dichroic mirror (DBS-1), passed through the microscope objective, hits the specimen (droplet, microsphere, multiple emulsion), recollected by the microscope objective, reflected back by the dichroic mirror to the off-axis parabolic reflector, reflected to 90°, and enters the detection region. In the detection path, first, the reflected fluorescence signal passes through a collimator to narrow the divergence of the signal. Then, the setup housed two arms, one for spectral studies of the reflected signal, and other for high-throughput signal information (intensity vs. time and wavelength selection by the dichroic beam splitter, DBS-2). For spectral studies, a flipping mirror (FM) was mounted right after the collimator to reflect the signal again at 90°, passed through a select-able density filter followed by a lens, a laser band-stop filter, an optical-fiber coupling lens, and a fiber connecting to the spectrometer. The spectrometer had a dynamic range from 300 nm to 800 nm with absolute operational mode from 400 nm to 700 nm. In the second detection arm comes a notch filter, to cut off the laser wavelength from the reflected signal and some optics to focus the signal. Then, the signal comes to detection region, which housed optics and electronics units. For optics, it included a dichroic beam splitter, filters, optics, and photo-multiplier tubes. The electronics section included shutters to select specific photo-multiplier tubes (PMT) for the detection and a National instrument data acquisition card (DAQ) coupled with a field-programmable gate array (FPGA) circuit for signal processing. A comprehensive illustration of the setup is provided in Figure 1.

### 2.2. Fabrication of Microfluidic Devices

Microfluidic devices were manufactured according to the conventional soft lithography technique [21]. In the first step, a pattern is transferred to commonly employed negative epoxy photoresin, namely SU-8 photoresist, previously coated on a silicon wafer, followed by exposure to ultraviolet (UV) light through the mask pattern. UV illumination leads to the polymerization of the photoresist located under the transparent regions of the mask. After development, the master mold is ready for the next step. In a second step, the PDMS (Polydimethylsiloxane) is first mixed with a cross-linking agent with a weight ratio of 10:1; the mixture is then degassed using a vacuum pump at room temperature, and the solution is poured onto the previously fabricated mold and placed in the oven for polymerization at 75 °C for 2 h. The block of PDMS is then removed from the mold; we thus obtain a replica of microchannels. In the third step, the PDMS block and the glass slide are treated with oxygen plasma for 20 s to enable their bonding and seal the microfluidic chip. The design of the microfluidic device for droplets generation contained two inputs; one input for the carrier oil and a second input for the dispersed phase, a main (square) channel with different cross sections, and an output for the collection of droplets in a Petri dish. The flow rates of the carrier oil ($Q_{c}$) and the dispersed phase ($Q_{d}$) were set using Nemesys syringe pumps (Cetoni GmbH, Korbußen, Germany).

The used carrier oil phase consisted of a fluorocarbon oil (HFE 7500, 3-ethoxy-dodecafluoro-2-trifluoromethyl-hexane, Inventec, Vincennes, France), with a density of 1.61 g/cm${}^{3}$ and thermal diffusivity of about $\kappa =3.6\times 10^{-8}$ m${}^{2}$/s; 2% Krytox surfactant was added in HFE 7500 oil in order to prevent droplets merging. The used fluorocarbon oil has the advantage of not inducing PDMS swelling and is also chemically inert and does not interact with droplet contents nor swell the microfluidic device substrates.

### 2.3. Temperature Measurements

We used a thermo-plate heating chamber (Tokai-Hit, Fujinomiya-shi, Japan) to control precisely the temperature of the whole microfluidic device (±0.1 °C). This device is equipped with two heating plates, one at the bottom and one at the top. The top plate incorporates an integrated glass heating element, which provides a uniform temperature distribution in the whole chamber. The heating device is also equipped with a feedback sensor mechanism that enables a real-time, precise sample temperature feedback temperature regulation. The heating chamber was placed on the microscope stage to measure the fluorescence of Rhodamine B droplets at different temperatures from 20 to 50 °C. To determine the dependence of the fluorescence intensity vs. temperature, we enclosed the entire microfluidic chip in the heating chamber. For each temperature value, we waited at least 10 min before recording the fluorescence intensity. Furthermore, in order to give enough time for the incoming microdroplets and HFE 7500 oil to reach the selected (target) temperature, a length of the tubing inlets of about L≃10 cm was enclosed in the heating chamber.

## 3. Results and Discussion

### 3.1. Experimental Demonstration of Anti-Stokes Fluorescence Emission of RhB Dye at the Droplets Interface

**Figure 2:**
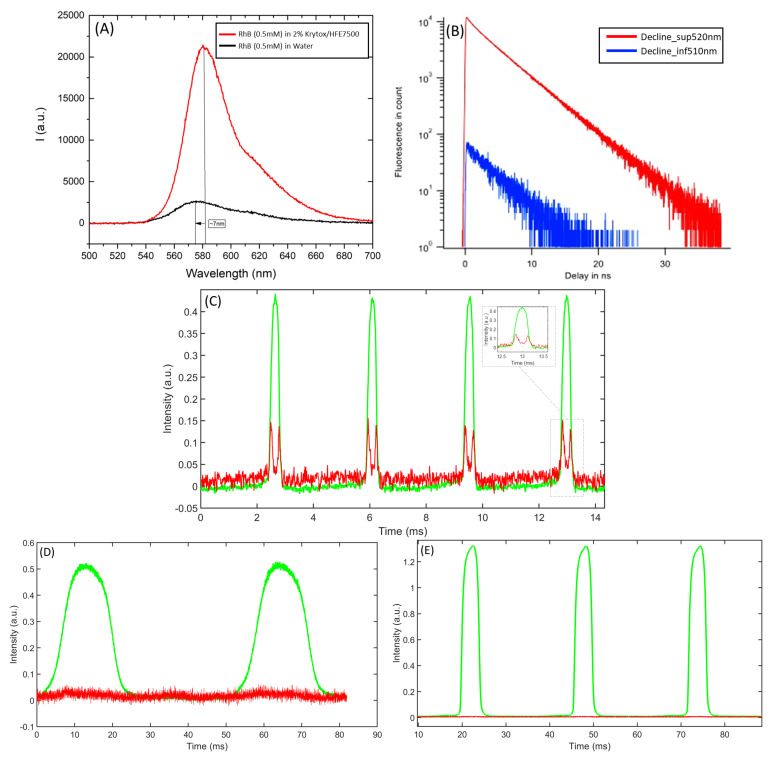
Rhodamine-B emission into different solvents and in different experimental conditions used in the experiment. (**A**) Fluorescence spectrum of RhB (Rhodamine-B) dissolved in water (Red) and in fluorocarbon oil with 2% wt. krytox surfactant (Black); (**B**) fluorescence lifetime graph of the RhB dissolved in krytox/fluorocarbon oil; (**C**) Fluorescence intensity vs. time plots of RhB in water droplets flowing in HFE-7500 fluorocarbon oil (containing krytox surfactant (2% wt.), each pulse represents the passage of a single droplet across the 532 nm excitation laser beam, and each droplet passage shows two distinct signals, one in green, which corresponds to standard fluorescence signal above 540 nm, but the red color curve recorded in the 500–520 nm range (below the excitation wavelength) is what makes the sensitivity of our system a new detection scheme; (**D**) fluorescence intensity vs. time plots of large RhB in water droplets flowing in HFE-7500 fluorocarbon oil without any surfactant; (**E**) fluorescence intensity of RhB in benzyl alcohol droplets vs. time in HFE-7500 fluorocarbon oil; (**D**,**E**) are used as control experiments where no blue-shifted emission signal is detected.

Figure 2A shows emission spectra of RhB in both water and HFE-7500 fluorocarbon carrier oil (2% wt. of Krytox), with maximum intensities around 582 nm and 575 nm, respectively. Figure 2B shows the fluorescence exponential decay of RhB in HFE oil vs. time, which was recorded on another dedicated setup described elsewhere [22,23]. The setup includes a 510 nm CW laser excitation and a notch filter cutting off wavelengths between 500 nm and 520 nm (approximately). The red curve (trace) in Figure 2B corresponds to the decline of fluorescence emission for wavelengths λ > 520 nm, whereas the blue trace corresponds to the decline of blue-shifted emission (λ < 510 nm). One can deduce from these measurements a fluorescence lifetime of about 4 ns for RhB dye in HFE oil, which is of the same order as the value found for RhB in water (1.7 ns) and corresponds indeed to a fluorescence emission [24]. The observed 7 nm shift between the two maxima should be attributed to the solvatochromism effect exhibited by many fluorescent dyes when dissolved in different solvents.

We present in Figure 2C the fluorescence intensity vs. time of RhB droplets (0.5 mM), with a mean diameter and velocity of approximately 33 μm and 6.3 cm/s, respectively. Droplets were carried by HFE-7500 fluorocarbon oil in a 30 μm × 30 μm microfluidic channel. We observe two types of photoluminescence signals. The first one (green) corresponds to the standard fluorescence signal of RhB, i.e., Stokes shifted emission, recorded for wavelengths above 540 nm, which are at higher wavelengths than the excitation’s. The second type of photoluminescence (red signal), detected in the range 500–520 nm, i.e., at smaller wavelengths than the excitation wavelength, corresponds to anti-Stokes emission of fluorescence of these droplets. Moreover, as shown in Figure 2C, the observed anti-Stokes photoluminescence from droplets is mainly localized at the interface of the droplets, whereas the standard (Stokes) fluorescence signal does not show any significant difference between the droplet interface and its bulk. This result suggests that anti-Stokes photoluminescence should be enhanced by the surrounding molecules of RhB molecules at this interface, i.e., either fluorocarbon oil molecules or/and krytox surfactant molecules.

In order to better understand the origin of such an effect, we recorded photoluminescence signals from droplets of RhB in water carried by HFE-7500 fluorocarbon oil alone (without krytox surfactant) and from droplets made of RhB in benzyl alcohol (BA). Results are shown in Figure 2D and Figure 2E, respectively. We notice an absence of a blue-shifted signal (red) in both cases. This result shows first that the detected blue-shifted photoluminescence (BSPL) signal corresponds definitely to a genuine anti-Stokes laser-induced fluorescence and not to an experimental artifact, e.g., an external noise in the 500–520 nm channel. It also shows that this phenomenon is enabled by the presence of krytox surfactant or/and the presence of RhB molecules inside the fluorocarbon oil phase. It’s worth noting that RhB molecules are highly soluble in Benzyl alcohol, which hinders the diffusion of RhB molecules into fluorocarbon oil. The size and droplet velocities were 103 μm and 1.9 cm/s, respectively, where other experimental conditions, such as surfactant concentration and excitation wavelength, were kept constant [14].

### 3.2. Effect of the Surfactant Droplets on Anti-Stokes Fluorescence Emission

**Figure 3:**
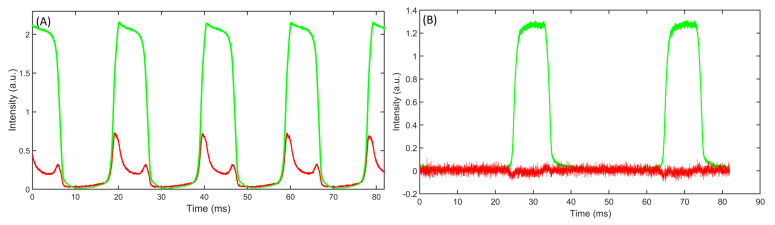
Effect of surfactant on the presence of anti-Stokes emission from RhB droplets flowing in a microfluidic channel. (**A**) Anti-Stokes and Stokes emission of rhodamine-B is present when droplets are in 2% Krytox surfactant; (**B**) Anti-Stokes emission (red) is practically not detected in the case of large droplets when using a non-electrically charged surfactant (KryJeffa) in HFE oil.

Figure 3 shows the intensity of ASF of large droplets (ø130 μm) made of RhB in water and flowing in HFE-7500 fluorocarbon oil with krytox surfactant and in HFE-7500 fluorocarbon oil with KryJeffa surfactant. In contrast with krytox surfactant, KryJeffa surfactant is made of a fluorocarbon polymer block linked to a hydrophilic PEG block and does not carry any electrical charge like krytox does.

We notice for large droplets stabilized with krytox that the maxima of both green and red signals are significantly shifted towards the left side of the peaks, i.e., towards the rear part of the droplet interface, giving hence to the photoluminescence signals a sawtooth-like shape (Figure 3A). Such an effect is usually observed in droplet microfluidics, and it is attributed to the well-known tip-streaming effect [25,26]. Moreover, when the used surfactant carries a negative electric charge, like krytox, the higher density of surfactant molecules at the droplet back interface (induced by the tip-streaming effect) leads, in turn, to an increase in the density of positively charged RhB molecules (at pH = 3) at this same area, driven by electrical interactions. This effect enables, hence, the probing of the distribution of RhB molecules inside the droplet and at its interface optically. We may notice also that the increase in photoluminescence signal at the rear part of the droplet interface is much more enhanced for anti-Stokes fluorescence (red signal) than for Stokes fluorescence (green signal). Interestingly, when using the non-ionic KryJeffa surfactant (Figure 3B), though the tip-streaming effect should still be active, we do not observe the sawtooth-like shape on the fluorescence (green) signal of RhB anymore. The missing of any noticeable asymmetry between the fluorescence intensity at the rear and the front parts of the droplets does not mean that there should be no difference between them when using Kryjeffa surfactant. It means only that there is no difference between the density of rhodamine molecules at the front and at the back of the droplet. This effect may be explained by the absence of electrostatic interaction between rhodamine B molecules and Kryjeffa surfactant molecules [14,25,26,27].

More interesting is the absence of the anti-Stokes (red) photoluminescence when KryJeffa surfactant is used. Furthermore, one may notice the approximately rectangular shape of the fluorescence intensity green peaks in Figure 3A, which indicates that droplets are more likely to have a rigid interface, which does not deform easily in the viscous flow. In contrast, without surfactant, as shown in Figure 3B, the shape of droplets’ fluorescence intensity peaks shows an approximate bell shape. This indicates that droplets, in this case, are more likely to adopt a soft interface, which deforms easily in the viscous flow. This may be explained by the increase in fluorescence intensity as the curved interface of the droplet moves more and more across the (still) laser spot before reaching a maximum value when the overlap between the laser footprint and the droplet is at its maximum. It’s worth noting that KryJeffa surfactant is a much more efficient surfactant than krytox surfactant due to the presence of the PEG hydrophilic block, which may also hinder the diffusion of RhB molecules to the HFE-7500 fluorocarbon oil phase. Based on these observations, it appears that anti-Stokes fluorescence is enabled when RhB molecules are rather in contact or surrounded by fluorocarbon oil molecules.

**Figure 4:**
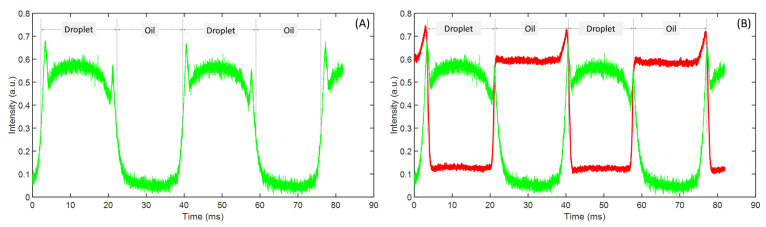
Emission of intense anti-Stokes signals from flowing droplets. (**A**) Stokes fluorescence intensity of large RhB in water droplets vs. time in HFE-7500 fluorocarbon oil stabilized by TBA-Krytox surfactant. (**B**) Both Stokes and anti-Stokes fluorescence intensities of RhB in water droplets (green signal) and fluorocarbon oil (red signal) in the presence of TBA-Krytox surfactant. One may remark that during the passage of the fluorocarbon oil plugs across the laser beam, an intense anti-Stokes fluorescence emission is detected.

Finally, we present in Figure 4 the result of an experiment where we used another surfactant, namely TBA-Krytox, for which the ionic charge of krytox molecules was neutralized by the addition of a strong base, which consists of tri-benzyl ammonium (TBA). It’s worth noting that when using such a surfactant, fluorocarbon oil turns pink-colored, indicating that rhodamine molecules diffuse easily from droplets to the continuous oil phase in the presence of this surfactant. The fluorescence pattern from this experiment is quite different from the previous ones. In particular, one observes that during the passage of the fluorocarbon oil plugs across the laser beam, a relatively intense ASF emission is also detected. This result confirms that the origin of the ASF signal should be tightly linked to the surrounding RhB molecules by HFE-7500 fluorocarbon oil molecules.

### 3.3. Effect of the Incident Laser Beam Power

Inspired by the results we obtained with RhB droplets and TB-krytox surfactant, we performed an experiment where RhB was solubilized directly in fluorocarbon oil, using 2% (wt.) of krytox in a single-phase microfluidic flow (no droplets), while the power of the excitation laser beam was varied from 0.5 mW to 3.5 mW, approximately.

**Figure 5:**
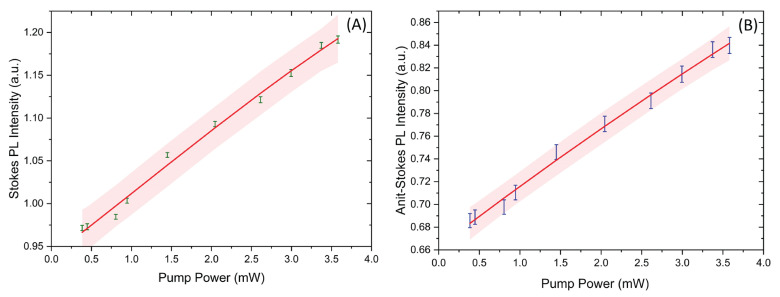
Variation of Stokes (**A**) and anti-Stokes (**B**) Fluorescence intensities of RhB in HFE-7500 fluorocarbon oil in a single phase microflow. The normalized rate of the linear increase in the intensity vs. the power of the excitation laser beam for both standard fluorescence and anti-Stokes fluorescence is found to be approximately 0.06 mW.

Results presented in Figure 5 show the intensity of both Stokes and anti-Stokes fluorescence vs. the power of the pumping laser. One can notice that both Stokes and anti-Stokes fluorescence signals increase linearly as the power excitation intensity increases, with practically the same normalized rate (quantum yield), that is, 5.7 ×$10^{-2}$/mW and 6.0 × $10^{-2}$/mW, respectively. Such a linear variation confirms a single photon absorption process which should be at the origin of the observed anti-Stokes and Stokes emission. Basically, such results may be easily understood according to simple theoretical considerations. Indeed, let us first consider a dye solution, dV=dA×dx an elementary volume, where dx represents its “thickness” along the x-direction parallel to an incident light beam, and dA its cross section. Let also I(x) be the intensity (per unit surface) of the incident light beam when it enters the elementary volume dV. According to the Beer–Lambert law, the change in I(x) through the thickness dx may be expressed as

(1) $$\frac{dI}{I}=-\epsilon Cdx,$$

where ϵ represents the extinction coefficient (or absorption coefficient) and *C* the concentration of the dye solution (assumed to be homogeneous). Integration of the previous differential equation leads to the expression of the intensity of the light beam across dV, which is $I\left(x\right)=I_{0}e^{-\epsilon Cx}$.

In principle, only a fraction of the light beam energy (absorbed locally by the dye) will be emitted as laser-induced fluorescence (LIF) in the related emission band. The ratio, Φ, of LIF intensity with respect to the absorbed light intensity is defined as the quantum yield (or efficiency) of the dye. Furthermore, the fluorescence quantum yield depends on many parameters such as the polarity of the solvent, dye concentration, temperature, or pH. For our study, RhB exhibits a high fluorescence quantum yield (Φ>0.5) at low concentrations ($10^{-4}$ to $10^{-6}$ M) [28,29,30], whereas at higher concentrations (>10${}^{-3}$ M), the quantum yield decreases rapidly to less than 0.1 [28,29,31]. Currently, it is widely accepted that at high concentrations in aqueous solutions, RhB molecules form dimers (due to strong electrostatic and dispersion interactions) with an equilibrium constant of about 2100 mol${}^{-1}$ (at 20 ${}^{{}^{\circ}}$C) [29]. Because dimers of RhB can only make a weak contribution to fluorescence (whereas they are capable of strong optical absorption), the fluorescence quantum yield of high-concentration aqueous RhB solutions can be strongly affected by dimerization. Within the frame of a single photon absorption process, the absorbed light intensity −dI×dA by the dye molecules confined in the volume dV should correspond to an emitted fluorescence intensity $dI_{F}$, which may be expressed as

(2) $$dI_{F}=-\Phi dIdA$$

Combining Equations (1) and (2) leads to

(3) $$\frac{dI_{F}}{dV}=\Phi \epsilon CI,$$

Hence, the intensity (per unit volume) of the fluorescence emitted by a dye solution increases linearly with (i) the intensity *I* of the light excitation, (ii) the concentration *C* of the dye solution, and (iii) the quantum yield Φ. Furthermore, applying Equation (3) and assuming that the absorption coefficient, ϵ, to be the same for both photoluminescence processes, one can conclude that the quantum yield should be practically the same for both Stokes emission and anti-Stokes emission, $\Phi_{s}$ and $\Phi_{as}$, respectively.

### 3.4. Dependence of Stokes and Anti-Stokes Fluorescence vs. Temperature in a Microfluidic Chamber

**Figure 6:**
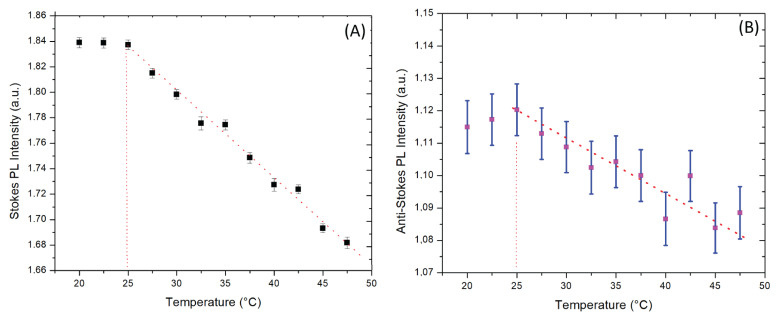
Variation of Stokes (**A**) and anti-Stokes (**B**) fluorescence intensities of RhB in fluorocarbon oil under 3.5 mW power illumination. We observe two temperature intervals: (i) below 25 °C, where the fluorescence intensity is practically constant, (ii) and above 25 °C, where the fluorescence intensity decreases linearly vs. temperature with a temperature sensitivity of about −0.4%/°C for Stokes fluorescence and −0.2%/°C for anti-Stokes fluorescence.

**Figure 7:**
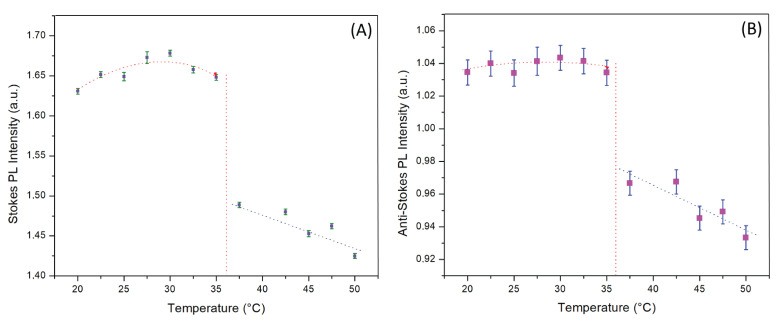
Variation of Stokes (**A**) and anti-Stokes (**B**) fluorescence intensities of RhB in fluorocarbon oil under 0.5 mW power illumination. We observe two temperature intervals: (i) below 36 °C, where the fluorescence intensity is more or less constant, (ii) and above 36 °C, where the fluorescence intensity decreases linearly vs. temperature with a temperature sensitivity of about −0.3%/°C.

Rhodamine B dye has been extensively studied for its highly sensitive fluorescence emission to temperature. We investigated in this work the dependence of anti-Stokes fluorescence of RhB in fluorocarbon oil, in the 20 °C to 50 °C temperature range, for two excitation power values: 3.5 mW and 0.5 mW. Preliminary results are presented in Figure 6 and Figure 7, respectively. As one can notice, both Stokes and anti-Stokes fluorescence intensities decrease as temperature increases, but with a noticeable difference with the regular linear decrease observed for Stokes fluorescence intensity vs. temperature [15,16]. In particular, one may remark, for instance, that for 3.5 mW power illumination, we observe two temperature intervals: (i) below 25 °C, for which the fluorescence intensity is more or less constant, and (ii) above 25 °C, for which the fluorescence intensity decreases linearly vs. temperature with a temperature sensitivity of about −0.4%/°C for Stokes fluorescence and −0.2%/°C for anti-Stokes fluorescence, as shown in Figure 6. One may also remark that the Stokes fluorescence linear decrease in the thermal sensitivity is approximately five times smaller than the reported literature data [15,16].

More interestingly, with a lower pumping laser power of 0.5 mW, we observe another transition for both Stokes and anti-Stokes of fluorescence intensities at a temperature of 36 °C, as shown in Figure 7. Below this temperature, both Stokes and anti-Stokes fluorescence intensities are practically constant, whereas above this temperature, fluorescence intensities start to decrease linearly vs. temperature with a thermal sensitivity S∼−0.3%/°C.

### 3.5. Suggested Origin of the Anti-Stokes Photoluminescence in Oil Phase: Low Thermal Conductivity of Surrounding Fluorocarbon Oil

Let us first summarize the main features of the fluorescence of RhB in fluorocarbon HFE-7500 oil.

- The intensity of anti-Stokes fluorescence may be relatively high and may represent more than 60% of the intensity of Stokes fluorescence, whereas in water solution, anti-Stokes fluorescence is very weak and represents less than 5% of the Stokes fluorescence;
- The wavelength of the observed anti-Stock fluorescence is smaller than the excitation wavelength, which means that excess energy of the emitted photons by this process should be provided by the thermal bath;
- When the temperature is increased, both Stokes and anti-Stokes Fluorescence intensities remain practically constant before a transition temperature appears, above which fluorescence starts to decrease linearly with a thermal sensitivity of about −0.4%/°C (Stokes) or −0.2%/°C (anti-Stokes);
- The transition temperature value reveals that to depend on the power of the incident excitation light, for an excitation power of 3.5 mW, the transition temperature is found to be about 25 °C, whereas for a smaller excitation power (0.5 mW), the transition temperature was found to be about 36 °C.

It’s worth noting that the thermal conductivity of HFE-7500 fluorocarbon oil (at 25 °C) is approximately one order smaller than the water’s thermal conductivity: 0.065 W/m·K for fluorocarbon oil and 0.614 W/m·K for water. We suggest that this large difference in thermal conductivities of the fluorocarbon carrier oil and of the water should be at the origin of the difference in fluorescence properties of RhB in the two solvents. In particular, the small value of thermal conductivity of HFE-7500 should enhance a local accumulation of heat and an increased temperature under the illuminated volume dV of the solution sample by the highly focused excitation laser beam. When a RhB molecule absorbs an incident photon, the excited electronic state ($S_{1}$) may interact and exchange energy with the surrounding medium phonons. After a number of interactions, the excited electronic state will re-emit a photon, which can possess higher energy than that of the excitation photon. In this type of process, the additional energy of the emitted photon by anti-Stokes fluorescence is provided by the heat of the surrounding medium, while the excitation light, which irradiates the dye molecules, has a longer wavelength (lower energy) than that of the maximum emission, molecules located at a higher vibration energy level, also termed as ’hot band’, can absorb these photons and reach the excited state. Coupling between excited state electron and thermal energy can occur also. Finally, the excited state can decay back to the ground state ($S_{0}$) and generate a fluorescence emission whose wavelength is shorter than that of the excitation light. Since the anti-Stokes luminescence process occurs at higher vibrational levels of the singlet ground state, it may prefer molecules with rich vibrational energy levels and is strongly temperature dependent due to the fact that the populations of higher vibrational energy levels are determined by Boltzmann distribution. Additionally, populations of higher vibrational energy levels are very limited, so effective anti-Stokes luminescence is usually observed in dye molecules with high quantum yield and molar extinction coefficient, such as Rhodamine B. We believe that hot-band absorption materials are very promising optical probes for diagnosis and detection in vivo [7]. Nevertheless, one important problem to be addressed is the relatively low luminescence efficiency of hot-band absorption materials. Droplet microfluidics can be used to address such a problem, as light and heat may be both confined inside droplets by total internal reflection (TIR) on the droplet interface. Hence, we think there is plenty of room for improvement and advancement in this domain.

As mentioned in the introduction section, the temperature sensitivity of fluorescence emission of Rhodamine B may be explained by the existence of a photoinduced reaction leading to the formation of a highly polar excited conformer called TICT [17,18,19]. In the case of rhodamine B, it is the xanthene group that rotates during isomerisation process [17,18], and since such a rearrangement is thermally activated, the rate of formation of the TICT state increases with temperature.

Finally, based on our results related to the observed transition temperatures vs. the power of pumping laser excitation, we think that though still preliminary, these results may give a new insight onto the coupling between hot-band absorption, anti-Stokes emission, and heat transport under different experimental conditions, such as flow velocity, viscosity, etc. We suggest that the temperature transitions observed at 25 °C and 36 °C should be related to the well-known thermal cooling effect [9,10], which is induced by anti-Stokes emission and is at the basis of optical refrigeration [10]. In fact, when a substance absorbs a photon and emits another one of greater energy, this leads to the cooling down of the temperature of this substance by the pumping of its thermal energy. Therefore, when the temperature of the bath is increased (by actuation on the bath heating system), the final change in fluorescence intensity, i.e., increase or decrease, of dye molecules would depend on the respective rates of both (i) the decrease in local temperature vs. time due to anti-Stokes emission and (ii) the rate of increase in temperature driven by the heat of the bath and the heat brought by the pumping laser. Therefore, when using a high power pumping laser (3.5 mW), one may reach a transition temperature of around 25 °C, which is smaller than the transition temperature observed when using a smaller power pumping laser (0.5 mW). This transition temperature should actually correspond to the temperature at which anti-Stokes optical cooling cannot compensate anymore for the increase in temperature due to the heat brought by both the bath heating system and the pumping laser.

## 4. Conclusions

We developed a broadband highly sensitive optofluidic detection system which enabled us to show the first experimental evidence of anti-Stokes fluorescence emission from rhodamine B droplets in flow. We showed in particular that the intensity of anti-Stokes fluorescence is relatively high and may represent more than 60% of the intensity of Stokes fluorescence, the wavelength of the observed anti-Stokes fluorescence is smaller than the excitation wavelength, which means that excess energy of the emitted photons by this process should be provided by the thermal bath (surrounding fluorocarbon oil). We also showed that when the temperature is increased, both Stokes and anti-Stokes fluorescence intensities remain practically constant before a transition temperature appears, above which fluorescence starts to decrease linearly with a thermal sensitivity of about −0.4%/°C (Stokes) or −0.2%/°C% (anti-Stokes). The transition temperature value appears to depend on the power of the incident excitation light: for an excitation power of 3.5 mW, the transition temperature was found to be about 25 °C, whereas for a smaller excitation power (0.5 mW), the transition temperature was found to be about 36 °C.

## Data Availability

Not applicable.
